# Determination of miR-373 and miR-204 levels in neuronal exosomes in Alzheimer’s disease

**DOI:** 10.55730/1300-0144.5484

**Published:** 2022-06-04

**Authors:** Elifcan TAŞDELEN, Erguvan Tuğba ÖZEL KIZIL, Sabiha TEZCAN, Eray YALAP, Ayşe Petek BİNGÖL, Nüket YÜRÜR KUTLAY

**Affiliations:** 1 Department of Medical Genetics, Faculty of Medicine, Ankara University, Ankara, Turkey; 2 Department of Medical Genetics, Şanlıurfa Training and Research Hospital, Şanlıurfa, Turkey; 3 Department of Psychiatry, Faculty of Medicine, Ankara University, Ankara, Turkey; 4 Department of Neurology, Faculty of Medicine, Ankara University, Ankara, Turkey; 5 Department of Neurology, Dr. Nafiz Körez Sincan State Hospital, Ankara, Turkey

**Keywords:** MiR-204, miR-373, Alzheimer’s disease, neuron derived exosomes, NLRP3 inflammasome, *TXNIP*, *P2X7R*, *GLP-1R*

## Abstract

**Background/aim:**

NLRP3 inflammasome activation has been known to be involved in the etiology and progression of Alzheimer’s disease (AD). Furthermore, AD and diabetes mellitus have common pathomechanisms. It has been shown that P2X7R whose expression is increased in brain tissues with AD and plays a role in the activation of NLRP3 inflammasome is suppressed by miR-373 in patients with osteoarthritis. Therefore, the question of whether the suppressive effect of miR-373 on NLRP3 may have a role in the pathophysiology of AD comes to mind. On the other hand, it is known that the miR-204 level increases in response to TXNIP, another NLRP3 inflammasome inducer with high expression in AD. In primary human islets, miR-204 reduces the expression of GLP-1R. It has been discovered that in vivo deletion of miR-204 is protective against diabetes by increasing GLP-1R and insulin secretion. Considering the relationship between miR-204 and TXNIP and the relationship of miR-204 with diabetes suggests investigating the effect of miR-204 on the inflammatory pathway in AD. Based on the common pathophysiological mechanisms between AD and diabetes and the reported changes related to NLRP3 inflammasome, we analyzed miR-373 and miR-204 in neuron-derived serum exosomes in this study. Neuron-derived exosomes in neurodegenerative diseases are considered to be better candidates for developing potential biomarkers.

**Materials and methods:**

The expression levels of miR-204 and miR-373 were investigated in neuron-derived serum exosomes obtained from 15 patients with mild AD, 18 with moderate AD, and 21 cognitively healthy individuals.

**Results and discussion:**

The miR-204 and miR-373 expressions were significantly decreased in both patient groups compared to the control group. Therefore, we suggest that miR-204 and miR-373 are potential biomarkers for AD. However, due to the preliminary nature of this study, further large-scale studies are needed to support our findings.

## 1. Introduction

The most common cause of age-related dementia worldwide is Alzheimer’s disease (AD), a neurodegenerative disorder [1]. Globally, one in 10 people over 65 live with AD, and its prevalence is expected to almost triple by 2050 [2,3]. Late-onset AD accounts for the majority of cases with the disease (>90%–95%). In its etiology, multiple genetic risk factors, as well as environmental factors are implicated. To date, mutations with low penetrance have been reported [4]. Although the best-known susceptibility gene is *APOE* [5], a large number of new genetic loci have been identified in genome-wide association studies associated with the risk of developing AD [6]. These include loci associated with immune response (*CLU, ABCA7*, and *CR1*) and lipid metabolism (*SORL1, PICALM*, and *CD2AP*) [7–12]. In recent years, with the widespread use of sequencing techniques, rare variants with lower frequency have been identified in some genes associated with AD; e.g., *TREM2* [12], *PLD3* [13], and *UNC5C* [14].

In vivo and in vitro studies show that amyloid β peptide and Tau protein are the main elements that form the pathogenesis of AD. Neurobiological mechanisms, such as amyloid hypothesis, cholinergic hypothesis, Tau phosphorylation, oxidative stress, and calcium homeostasis are considered in the etiopathogenesis of AD [15]. There is also evidence in the literature that neuroinflammatory changes are involved in the etiology and progression of the disease [16].

miRNAs have important effects on the posttranscriptional regulation of gene expression, and although they do not encode proteins, they have become the focus of attention due to their potential as biomarkers in the diagnosis, monitoring and treatment of various diseases. miRNA expression changes observed in many diseases, including AD have been associated with the pathogenesis of the disease [17].

Exosomes are extracellular vesicles that play important roles in the signal transduction and transport of molecules. The evaluation of content carried by exosomes has brought up their potential to be used in the monitoring of diseases. It is anticipated that they may provide a great advantage in neurodegenerative diseases, such as AD, which requires a definitive diagnosis and invasive procedures [18,19].

Recently, the activation of NLR family pyrin domain-containing 3 (NLRP3) inflammasome with amyloid β (Aβ) has been shown to play a critical role in the pathogenesis of AD and provide the basis for IL-1β maturation and subsequent inflammatory events [20]. The expression of P2X purinoceptor 7 (P2X7R) around the amyloid plaque has been shown to increase in *APP* (amyloid precursor protein) mutant transgenic mice having high APP expression. After the in vivo inhibition of P2X7R, the number of hippocampal amyloid plaques decreased significantly in mouse models [21]. Zhang et al. showed that miR-373 suppressed the P2X7R expression by binding to the 3’UTR of P2X7R in patients with osteoarthritis and reduced inflammation and chondrocyte proliferation in these patients via suppressing the inflammatory factors such as IL-6 and IL-8 [22]. On the other hand, Sorensen et al., who obtained the miRNA profile from the cerebrospinal fluids of patients with AD found no significant change in miR-373 expression levels compared to the control group [23]. However, the expression of miR-373 in neuron-derived exosomes from serum has not been analyzed until now. In the current study, considering effect of miR-373 on P2X7R, the expression level of miR-373 in the neuron-derived exosomes of patients with AD was examined.

Previous studies have shown that the binding of thioredoxin-interacting protein (TXNIP) to NLRP3 is significant for inflammasome activation and insulin resistance [24]. In many neurodegenerative diseases, including AD, the level of TXNIP expression has been shown to increase [25,26]. Amelioration in Tau phosphorylation has been observed as a result of the inhibition of TXNIP and NLRP3 inflammasome [27]. Jo et al. described TXNIP as the upstream regulator of miR-204. In response to TXNIP, the miR-204 level increased. miR-204 was found to be lower in beta cell-specific TXNIP knockout mice [28]. On the other hand, the presence of Type 2 diabetes mellitus (DM) increases the risk of AD [29]. The mechanism underlying this relationship is deficiencies in insulin and glucagon-like peptide 1 (GLP-1) signal. GLP-1, which is expressed from the soma and dendrites of large neurons, shows a neuroprotective effect against the toxic activities of Aβ1-42. It is known that the cells of the hippocampus and the hypothalamus highly express glucagon-like peptide 1 receptor (GLP-1R) [30, 31]. Jo et al. showed that miR-204 suppressed GLP-1R expression in primary mouse and human pancreatic islets by targeting the 3’UTR of GLP-1R [28]. In a study by Solensen et al., there was no significant change in miR-204 expression in the cerebrospinal fluids of patients with AD compared to the control group [23]. Nevertheless, based on the above-mentioned studies, we investigated miR-204 expression in the neuron-derived exosomes of patients with AD in the current study.

According to the literature, there are many studies related to miRNAs in neurodegenerative diseases, including AD. It is known that the expression changes in the detected miRNAs contribute to the pathogenesis of AD from different aspects. In a study, low miR-153 levels were found to contribute to Aβ accumulation in individuals with sporadic AD [32]. Aβ plaques result from an imbalance between the production and degradation of amyloid peptides. Increased levels of miR-128 impair Aβ clearance by targeting lysosomal enzymes in sporadic Alzheimer’s patients [33]. In another study, it has been observed that the increase of miR-188-5p in primary hippocampal neurons reduces synaptic and cognitive losses [34]. miR-138, whose increased expression is observed in Alzheimer’s patients, has also been shown to induce Tau hyperphosphorylation by suppressing GSK-3β (glycogen synthase kinase-3b) activity [35]. Furthermore, miR-137 has been shown to reduce Aβ-induced neurotoxicity via suppressing the NF-kB pathway[36].

In recent studies, the diagnostic yield of inflammatory miRNAs in AD has been investigated. For instance, Souza et al. detected the downregulation of miR-9-5p in whole blood samples of AD patients compared to the control group [37]. In another study, it has been shown that the plasma levels of miR-17-5p, miR-21-5p, and miR-126-3p in a cohort of AD patients were upregulated compared to healthy controls [38]. Here, we focused on the pathomechanism of NLRP3 inflammasome and the common mechanisms of AD with diabetes. As noted above, we chose miR-373 and miR-204 as a preliminary study, based on the knowledge of previous studies that mentioned the expression change of P2X7R and TXNIP shown in AD and the regulatory effect of miR-373 and miR-204 on them. We planned to conduct a study on the exosomes of neural origin considering that it would provide more accurate information related to the pathogenesis of AD different from the above-mentioned studies in methodological terms. In addition, it was thought that the use of exosomes obtained from peripheral blood could become a routine procedure in the diagnosis and follow-up of this patient group. Furthermore, exosomes have therapeutic potential since miRNAs can be modified according to the detected expression changes and sent to the target.

## 2. Materials and methods

### 2.1 Participants

This study was approved by the ethics committee of the university and all procedures were undertaken in accordance with the latest version of the Declaration of Helsinki. Informed consent was obtained from all participants after a detailed explanation of the experimental procedure. The competencies of the volunteers in the patient group were evaluated by a neurologist and a psychiatrist. For those who were found to be incompetent, the consent was obtained from the legal representatives of the patients.

Fifteen patients with mild AD (Mi-AD), 18 with moderate AD (Mo-AD) and 21 older healthy volunteers were included in the study. The patients were evaluated at the geriatric psychiatry and neurology departments of a university hospital. Patients who were diagnosed with probable AD according to the diagnostic criteria of the National Institute of Neurological and Communicative Disorders and Stroke-Alzheimer’s Disease and Related Disorders Association [39] and who were compatible with the mild and moderate stages according to the Clinical Dementia Score (CDS) [40] were included in the study. None of the patients had any family history related to AD, and all were aged ≥60 years.

All the patients underwent MRI scans which were routinely performed for clinical diagnoses. The Scheltens’ scale for medial temporal atrophy was used to evaluate the level of medial temporal atrophy (hippocampal atrophy-HA) as a biomarker in the patients [41]. In addition, since the patients’ vascular brain lesions would make the interpretation of the findings difficult, especially due to eliminating possible accompanying vascular dementia, only those who scored 0 (no white matter lesion) or 1 (one or two white matter lesions with no tendency to merge) in the Fazekas scale were included in the sample [42]. The Turkish version of the Mini-Mental State Examination was administered to both the control and AD groups [43]. The detailed information of all the participants is given as supplemental data.

The exclusion criteria of the study were having another neurological disease that could impair cognitive evaluation (ischemic or hemorrhagic cerebrovascular disease, mental retardation, epilepsy, Parkinson’s disease, non-Alzheimer’s type of dementia, motor neuron disease, alcohol and substance dependence, etc.), advanced stage AD (CDS = 3), DM, and Fazekas scale score > 1.

The control group was selected from cognitively healthy volunteers. The exclusion criteria for this group were having a neurological disease that could impair cognitive assessment and the presence of a DM diagnosis.

### 2.2 Collection of samples from participants

Twenty mL of peripheral blood was collected from each participant with a sterile syringe and then transferred to serum separator tubes. It was allowed to clot for 30–45 min at room temperature. After coagulation, it was centrifuged at 500 g for 10 min. Then, 5 mL serum was taken from the supernatant portion with a Pasteur pipette. The serums were stored in aliquots at −80 **°C until use** [44].

### 2.3 Isolation of total exosomes and L1CAM-positive neuron-derived exosomes

The serum sample was thawed at 4 °C. Two-hundred μL of the precipitation buffer A solution (miRCURY Exosome Serum/Plasma Kit, Qiagen, catalog no: 76603) was added to 0.5 mL serum and vortexed for 5 s. The mixture was incubated at 2–8 °C for 60 min. Then, it was centrifuged at 1500 g for 30 min at 20 °C. The remaining supernatant was discarded. Two hundred and seventy **μ**L of resuspension buffer was added to the tube containing the pellet and resuspended by vortexing. A final volume of ~300 μL was obtained.

Two μg (4 μL) of mouse antihuman CD171 (L1CAM, neural adhesion protein) biotinized antibody (Thermo Fisher) and 100 μL 3% blocker BSA were added to 250 μL suspension of exosomes obtained by exosome isolation [45]. The sample was incubated in a rotator mixer for 60 min at 4 **°**C. After incubation, 15 μL Streptavidin UltraLink Resin (Thermo Fisher) and 40 μL 3% blocker BSA were added. Following incubation for 30 min at 20 °C, the sample was centrifuged at 3000 g for 10 min at 4 **°**C. The supernatant was discarded. To remove neuronal exosomes trapped in resin, 200 μL of 0.1M glycine-HCl (pH:3) was added to the pellet and mixed for 30 s with a quick vortex. It was centrifuged at 4500 g for 10 min at 4 °C, and then the supernatant was removed, and neutralization was achieved by adding 15 μL of 1M TRIS-HCl (pH:8). Phosphate-buffered saline was added until the suspension reached a volume of 300 μL.

### 2.4 miRNA isolation from L1CAM-Positive neuronal exosomes

miRNA was extracted using spin column chromatography (miRNeasy Serum/Plasma Kit, Qiagen catalog no: 217184). Five mL of QIAzol Lysis Reagent was added to 300 μL of sample and mixed by vortexing. Twenty **μ**L of the carrier RNA solution was added, and the sample was incubated for 5 min at room temperature. One mL of chloroform was added, and the tube was tightly capped and vortexed vigorously. After incubation for 2–3 min at room temperature, it was centrifuged at 1500 g for 15 min. The upper aqueous phase was transferred to a new collection tube. Then, 100% ethanol was added at 1.5 times of the volume of the sample. It was mixed thoroughly by pipetting. Seven hundred **μ**L of the sample was added into the RNeasy MinElute spin column, which was then centrifuged at ≥8000 g for 15 s at room temperature. This step was repeated until the sample was finished. Seven hundred**μ**L of the buffer RWT was added to the RNeasy MinElute spin column, capped, and centrifuged at 8000 g for 25 s. The bottom part was discarded. Five hundred μL of the buffer RPE was added to the RNeasy MinElute spin column, capped, and centrifuged at 8000 g for 25 s, and the bottom part was discarded. Five hundred μL of 80% ethanol was added to the RNeasy MinElute spin column. The lid was closed and centrifuged at ≥8000 g for 2 min 15 s. The bottom and collecting tubes were discarded. The RNeasy MinElute spin column was placed in a new 2 mL collection tube. The spin column was opened and centrifuged at full speed for 5 min to dry the membrane. The bottom and collecting tubes were discarded. The RNeasy MinElute spin column was placed in a new 1.5 mL collection tube. Fourteen μL of RNase-free water was directly placed in the center of the spin column membrane. It was slowly capped and centrifuged for 1 min 15 s at full speed to obtain miRNA.

### 2.5 cDNA synthesis and real-time PCR

cDNA synthesis was performed with the miRCURY LNA Reverse Transcription (RT) Kit, Qiagen (catalog no: 339340) from the obtained miRNAs. To obtain cDNA, each miRNA sample was incubated for 60 min at 42 °C in the presence of 5 × miRCURY RT Reaction Buffer, RNase-free water, 10x miRCURY RT Enzyme Mix (for miR-204-5p, miR-373-3p, U6 snRNA (for endogenous control). RT was inactivated within 5 min at 95 °C, and the cDNA samples were cooled at 4 °C. cDNA samples for real-time PCR were mixed with 2x miRCURY SYBR Green Master Mix, PCR primer mix, and RNase-free water (mirCURY LNA RT Kit, Qiagen catalog no:339340). The plates were run on the QIAGEN’s real-time PCR cycler, Rotor-Gene Q.

### 2.6 Evaluation of results and statistical analysis

Reactions for both the target miRNA (miR-204 and miR-373) and endogenous control (U6 snRNA) were performed against a negative template control in each case and control. U6 snRNA was used as a reference for normalization. The miR-204, miR-373 and U6 snRNA cycle threshold (Ct) values of each case and control were determined by averaging the Ct values emerging from their two reactions. The Ct values for U6 snRNA, miR-204, and miR-373 of the patient and control groups are given as supplemental data. Using the obtained data, miRNA expression changes were calculated with the 2^−^^ΔΔ^^Ct^ (Livak) method as previously described [46].

Data obtained from the study were summarized using descriptive statistics. For this purpose, the mean ± standard deviation [median (minimum-maximum)] values were calculated for the miR-204 and miR-373 levels. The compatibility of the measurements obtained from the groups to the normal distribution was examined with the Kolmogorov-Smirnov test, and the homogeneity assumption of the group variances was examined with the Levene test. Since the data showed a normal distribution, the significance of the difference between the groups was examined with a one-way analysis of variance (ANOVA), and the group from which the difference originated was examined with the Dunnet test. p < 0.05 was considered statistically significant.

## 3. Results

A sample consisting of 15 patients with mild AD, 18 with moderate AD, and 21 healthy controls was investigated. The participants’ demographic and clinical information are given in Table 1. The Ct1, Ct2, and average Ct values of each participant are given in supplemental data. The mean Ct values for U6 snRNA, miR-204, and miR-373 are shown in Table 2, and the average delta Ct values which were calculated using the mean Ct values are presented in Table 3.

The fold change of gene expressions shows the results found with the 2 ^−^^ΔΔ^^Ct^ (Livak) method. As shown in Table 4a, the expression levels were observed as 0.14-fold for hsa-miR-204 in the mild AD group and 0.04-fold in the moderate AD group compared to the control group. For hsa-miR-373, the fold change was 0.27 in the mild AD group and 0.16 in the moderate AD group compared to the control group. In Figure, the fold change values of the mild and moderate AD groups for miR-204 and miR-373 are shown graphically compared to the control group. After U6 snRNA normalization, the “fold change” values calculated for both miRNAs in the mild and moderate AD groups were found to be decreased compared to the control group.

p values were determined by applying Student’s t-test to the 2 ^−^^ΔΔ^^Ct^ values of each miRNA in the AD and control groups (Table 4b). For both miRNAs, the expressions were reduced in the moderate AD group compared to the mild AD group. While the decrease in the miR-204 expression in the mild and moderate AD groups was statistically significant compared to the control group (p = 0.000659 and 0.000051, respectively), the difference between the mild and moderate AD groups was not statistically significant (p = 0.131). Similarly, the decrease in the miR-373 expression in mild and moderate AD groups was statistically significant compared to the control group (p = 0.0224 and 0.00325, respectively), but the difference between the mild and moderate AD groups was not statistically significant (p = 0.546).

## 4. Discussion

Studies carried out to date, have shown that exosomes in the central nervous system (CNS) carrying mRNA, noncoding RNA, and DNA are involved in many important processes such as intercellular communication, trophic support of neurons, antigen presentation, and synaptic plasticity [47]. Since it is considered that exosomes reflect the inside of the cell better than other body fluids in terms of their nucleic acid and protein contents, they have recently been the focus of interest in studies aiming to reveal the pathogenesis of neurodegenerative diseases. In addition, it has been shown that exosomes are more advantageous than cell-free RNAs due to the amount of RNA content [48]. There are some biomarker studies using neuron-derived exosomes that express CD171 as a surface marker to reflect CNS diseases more specifically. It has been shown that autolysosome protein levels in neuron-derived exosomes from the blood samples of AD cases are higher than in the control group, which may be a predictor of AD before clinical onset [49]. Furthermore, Walsh et al. found that miR-212 and miR-132 levels in neuron-derived exosomes were lower in the plasma samples of patients with AD than in the control group [50]. In another miRNA profile study comparing the total plasma extracellular vesicles population and neuron-derived extracellular vesicles, it was observed that the levels of miR-23a-3p, miR-223-3p, and miR-190a-5p increased whereas the miR-100-3p levels decreased [51]. However, the use of plasma as a source of exosomes creates difficulties due to the coagulation factors found in the plasma content which may coprecipitate with exosomes. Therefore, additional procedures are required, such as the use of EDTA or heparin to reduce coagulation and obtain pure exosomes [52]. We predicted that the use of serum instead of plasma would be more advantageous since it does not require an additional procedure from a methodological point of view. In addition, although there are studies concerning the altered expression of miRNAs in exosomes isolated from plasma in patients with AD as mentioned above, there is no information in the literature regarding the expression changes of miRNAs in neuron-derived exosomes from the serum samples of this patient group. For these reasons, in the current study, neuron-derived exosomes were extracted using the anti-CD171 (L1CAM) antibody from the serum samples of 15 mild and 18 moderate AD cases and 21 volunteers without any cognitive deficit. Then, total miRNAs were obtained from the neuron-derived exosomes, and the miR-204 and miR-373 expressions were analyzed in the AD and control groups. The expression of both miRNAs was found to be significantly lower in the mild and moderate AD groups than in the controls. However, the difference between the mild and moderate AD groups was not statistically significant for miR-204 or miR-373.

In a study examining the postmortem brain tissues of a transgenic mouse model of AD, it was shown that the expression and function of P2X7R were upregulated [21]. In patients with osteoarthritis, miR-373 has been shown to suppress P2X7R expression by binding to its 3’UTR and decrease inflammation and chondrocyte proliferation in these patients [22]. Sorensen et al., who obtained the miRNA profile from the cerebrospinal fluids of patients with AD reported no significant change in miR-373 expression compared to the control group [23]. To date, no study has associated miR-373 with AD. We consider that the lower miR-373 levels of our patients with AD compared to the control group indicate that P2X7R could not be suppressed in these patients, and as a consequence the NLRP3 inflammasome pathway was activated, contributing to neuroinflammation. This mechanism can be more clearly demonstrated in further studies investigating the expression of P2X7R in AD and control groups and comparing the results with the miR-373 expression data.

A review of the literature suggests an increase in the expression of miR-204 in patients with AD compared to the control group based on publications reporting an increase in miR-204 in response to TXNIP in diabetic patients or decreased expression of GLP-1R by miR-204 [28]. However, our results revealed a low level of miR-204 expression in both AD groups compared to the control group. There are very few studies on this subject in the literature. This finding can be explained by miRNAs having a large number of unspecified target genes and the presence of factors that control their expression which have not yet been fully identified. However, there are some studies supporting our results. In a study examining frontotemporal dementia (FTD), a patient group carrying mutation in one of the three genes (*C9orf72, GRN*, and *MAPT*), presymptomatic individuals carrying mutations, and a healthy control group that did not carry any mutation were evaluated, and the expression levels of miR-204 in exosomes obtained from cerebrospinal fluid were shown to be lower in the symptomatic patients compared to the other groups [53]. As a result of the in silico analysis, the authors reported that *HRK*, which encodes the apoptosis activator HARAKIRI, was one of the target genes of miR-204 [53,54]. Considering the common neurodegeneration mechanisms in FTD and AD, the lower miR-204 levels in our AD group may have upregulated *HRK* and caused apoptosis and neurodegeneration. In another study, the expression levels of miR-204 in the hippocampus of *APP/PS1* mutant transgenic Alzheimer’s mouse models were found to be low. After the administration of lentivirus infusion over-expressing miR-204 to the same mice, it was shown that the amyloid plaque burden of mice was reduced, and memory loss was improved [55]. The compatibility of our results with the studies mentioned above shows the effectiveness of the use of neuron-derived exosomes as biomarkers for the early diagnosis, follow-up and treatment of neurodegenerative diseases.

In our study, although the miR-204 and miR-373 expression levels were found to be lower in the moderate AD group compared to the mild AD group, this finding can be interpreted as progressing neurodegeneration in these patients. However, in order to support this inference, it is necessary to conduct a study with a larger sample size. Although the use of neuron-derived exosomes is predicted to provide increased specificity in understanding the pathophysiology of CNS diseases, such as AD, this requires a comparison of data obtained from studies examining the miRNA expression levels of total exosomes isolated from serum samples.

It would be ideal to carry out a transcriptome analysis and analyze the expression profile of miRNAs in patients with AD of different stages and in healthy volunteers from different age groups, but this would increase the cost tremendously. Therefore, the current study was designed as preliminary research. In future studies, it is planned to expand the sample size and conduct extensive research to determine the target genes of these miRNAs.

There is not yet a specific treatment for AD. Today, studies on biomarkers that can be detected in the early stages of the disease have gained importance. Instead of biomarkers, such as total Tau, phosphorylated Tau-181, and Amyloid β proteins in the cerebrospinal fluid [56, 57], which are obtained by invasive protocols, the plasma phosphorylated Tau-217 is a promising alternative [58, 59]. Similar studies seeking plasma or serum biomarkers can offer new horizons for both the early diagnosis and potential therapeutic targets for AD with increasing prevalence.

## Supplementary Information



## Figures and Tables

**Figure:**
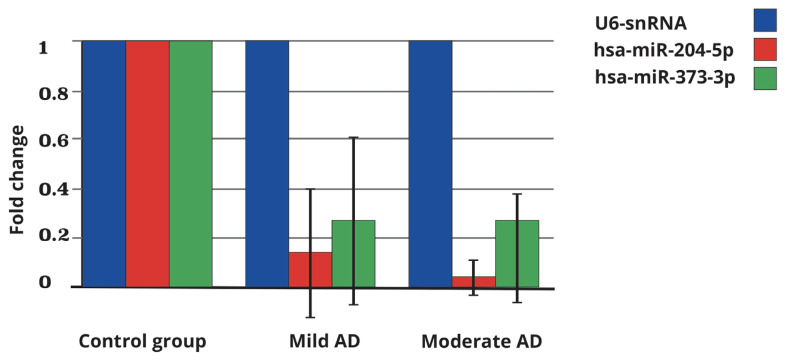
Graphical representation of the fold change values of the mild and moderate stage AD groups for miR-204 and miR-373 compared to the control group.

**Table 1:** Sociodemographic and clinical characteristics of the participants. Mi-AD: Mild Alzheimer’s disease group, Mo-AD: Moderate Alzheimer’s disease group, HC: Healthy control group, M: Male, F: Female, MMSE: Mini-Mental State Examination, SD: Standard deviation

	n	Gender (M: F)	Mean age ± SD	Mean years of education ± SD	Mean MMSE score ± SD
**Mi-AD**	15	6:9	71.40 ± 7.76	8.27 ± 5.42	23.7 ± 2.8
**Mo-AD**	18	4:14	76.94 ± 9.10	6.89 ± 5.42	18.3 ± 4.63
**HC**	21	12:9	66.38 ± 6.07	Not documented	28.3 ± 1.32

**Table 2:** Average Ct values for U6 snRNA, miR-204, and miR-373. Ct: Cycle threshold, Mi-AD: Mild Alzheimer’s disease group, Mo-AD: Moderate Alzheimer’s disease group, HC: Healthy control group.

	Mean Ct values			Standard deviation			P value
	HC	Mi-AD	Mo-AD	HC	Mi-AD	Mo-AD	
U6 snRNA	20.84	24.74	21.49	1.542940	1.554326	1.634782	<0.001
hsa-miR-204	21.52	28.30	26.90	2.238471	2.336613	2.036743	<0.001
hsa-miR-373	24.85	30.65	28.13	1.118668	1.896381	2.386306	<0.001

**Table 3:** Average delta Ct values for U6 snRNA, miR-204, and miR-373. Mi-AD: Mild Alzheimer’s disease group, Mo-AD: Moderate Alzheimer’s disease group, HC: Healthy control group, Ct: Cycle threshold.

	Mean delta (Ct) [Ct (AD group)–Ct (HC)]			Standard deviation		
	HC	Mi-AD	Mo-AD	HC	Mi-AD	Mo-AD
U6 snRNA	0.00	0.00	0.00	0.000000	0.000000	0.000000
hsa-miR-204	0.68	3.56	5.40	1.959049	2.018526	1.867945
hsa-miR-373	4.01	5.91	6.64	1.504755	1.043574	1.289807

**Table 4a:** Fold change (2’ Ct) values for miR-204 and miR-373 compared to the control group. Mi-AD: Mild Alzheimer’s disease, Mo-AD: Moderate Alzheimer’s disease, Ct: Cycle threshold.

	Fold change (2^−ΔΔCt^) (compared to the control group)	
	Mi-AD group	Mo-AD group
U6 snRNA	1.00	1.00
hsa-miR-204	0.14	0.04
hsa-miR-373	0.27	0.16

**Table 4b:** p values of the miR-204 and miR-373 expression levels in AD groups compared to the control group. AD: Alzheimer’s disease, Mi-AD: Mild Alzheimer’s disease group, Mo-AD: Moderate Alzheimer’s disease group.

	p-values (compared to the control group)	
	Mi-AD	Mo-AD
U6 snRNA	N/A	N/A
hsa-miR-204	0.000659	0.000051
hsa-miR-373	0.0224	0.00325

## References

[1] Prince M Wimo A Guerchet M Ali G Wu Y The global impact of dementia World Alzheimer Report 2015 1 82

[2] Mandrekar-Colucci S Landreth GE Microglia and inflammation in Alzheimer’s disease CNS & Neurological Disorders-Drug Targets (Formerly Current Drug Targets-CNS & Neurological Disorders) 2010 9 2 156 167 10.2174/187152710791012071 PMC3653290 20205644

[3] Hebert LE Weuve J Scherr PA Evans DA Alzheimer disease in the United States (2010–2050) estimated using the 2010 census Neurology 2013 80 19 1778 1783 10.1212/WNL.0b013e31828726f5 23390181 PMC3719424

[4] Naj AC Schellenberg GD Consortium AsDG Genomic variants, genes, and pathways of Alzheimer’s disease: an overview American Journal of Medical Genetics Part B: Neuropsychiatric Genetics 2017 174 1 5 26 10.1002/ajmg.b.32499 PMC6179157 27943641

[5] Mahley RW Apolipoprotein E: cholesterol transport protein with expanding role in cell biology Science 1988 240 4852 622 630 10.1126/science.3283935 3283935

[6] Wightman DP Jansen IE Savage JE Shadrin AA Bahrami S A genome-wide association study with 1,126,563 individuals identifies new risk loci for Alzheimer’s disease Nature genetics 2021 53 9 1276 1282 10.1038/s41588-021-00921-z 34493870 PMC10243600

[7] Shao W Peng D Wang X Genetics of Alzheimer’s disease: From pathogenesis to clinical usage Journal of Clinical Neuroscience 2017 45 1 8 10.1016/j.jocn.2017.06.074 28869135

[8] Wu Z-C Yu J-T Li Y Tan L Clusterin in Alzheimer’s disease Advances in clinical chemistry 2012 56 155 73 10.1016/b978-0-12-394317-0.00011-x 22397031

[9] Harold D Abraham R Hollingworth P Sims R Gerrish A Genome-wide association study identifies variants at CLU and PICALM associated with Alzheimer’s disease Nature genetics 2009 41 10 1088 10.1038/ng.440 19734902 PMC2845877

[10] Jin C Liu X Zhang F Wu Y Yuan J An updated meta-analysis of the association between SORL1 variants and the risk for sporadic Alzheimer’s disease Journal of Alzheimer’s Disease 2013 37 2 429 437 10.3233/JAD-130533 23948893

[11] Chen H Wu G Jiang Y Feng R Liao M Analyzing 54,936 samples supports the association between CD2AP rs9349407 polymorphism and Alzheimer’s disease susceptibility Molecular neurobiology 2015 52 1 1 7 10.1007/s12035-014-8834-2 25092125

[12] Wang Z Lei H Zheng M Li Y Cui Y Meta-analysis of the association between Alzheimer disease and variants in GAB2, PICALM, and SORL1 Molecular neurobiology 2016 53 9 6501 6510 10.1007/s12035-015-9546-y 26611835

[13] Cruchaga C Karch CM Jin SC Benitez BA Cai Y Rare coding variants in the phospholipase D3 gene confer risk for Alzheimer’s disease Nature 2014 505 7484 550 554 10.1038/nature12825 24336208 PMC4050701

[14] Wetzel-Smith MK Hunkapiller J Bhangale TR Srinivasan K Maloney JA A rare mutation in UNC5C predisposes to late-onset Alzheimer’s disease and increases neuronal cell death Nature medicine 2014 20 12 1452 1457 10.1038/nm.3736 PMC4301587 25419706

[15] Sanabria-Castro A Alvarado-Echeverría I Monge-Bonilla C Molecular pathogenesis of Alzheimer’s disease: an update Annals of neurosciences 2017 24 1 46 54 10.1159/000464422 28588356 PMC5448443

[16] Heneka MT Carson MJ El Khoury J Landreth GE Brosseron F Neuroinflammation in Alzheimer’s disease The Lancet Neurology 2015 14 4 388 405 10.1016/S1474-4422(15)70016-5 25792098 PMC5909703

[17] Wang M Qin L Tang B MicroRNAs in Alzheimer’s disease Frontiers in genetics 2019 10 153 10.3389/fgene.2019.00153 30881384 PMC6405631

[18] Janas AM Sapoń K Janas T Stowell MH Janas T Exosomes and other extracellular vesicles in neural cells and neurodegenerative diseases Biochimica et Biophysica Acta (BBA)- Biomembranes 2016 1858 6 1139 1151 10.1016/j.bbamem.2016.02.011 26874206

[19] Kanninen KM Bister N Koistinaho J Malm T Exosomes as new diagnostic tools in CNS diseases Biochimica et Biophysica Acta (BBA)-Molecular Basis of Disease 2016 1862 3 403 410 10.1016/j.bbadis.2015.09.020 26432482

[20] Halle A Hornung V Petzold GC Stewart CR Monks BG The NALP3 inflammasome is involved in the innate immune response to amyloid-β Nature immunology 2008 9 8 857 10.1038/ni.1636 18604209 PMC3101478

[21] Parvathenani LK Tertyshnikova S Greco CR Roberts SB Robertson B P2X7 mediates superoxide production in primary microglia and is up-regulated in a transgenic mouse model of Alzheimer’s disease Journal of Biological Chemistry 2003 278 15 13309 13317 10.1074/jbc.M209478200 12551918

[22] Zhang W Zhong B Zhang C Luo C Zhan Y miR-373 regulates inflammatory cytokine- mediated chondrocyte proliferation in osteoarthritis by targeting the P2X7 receptor FEBS open bio 2018 8 3 325 331 10.1002/2211-5463.12345 PMC5832977 29511609

[23] Sørensen SS Nygaard A-B Christensen T miRNA expression profiles in cerebrospinal fluid and blood of patients with Alzheimer’s disease and other types of dementia–an exploratory study Translational neurodegeneration 2016 5 1 6 10.1186/s40035-016-0053-5 26981236 PMC4791887

[24] Zhou R Tardivel A Thorens B Choi I Tschopp J Thioredoxin-interacting protein links oxidative stress to inflammasome activation Nature immunology 2010 11 2 136 140 10.1038/ni.1831 20023662

[25] Su CJ Feng Y Liu TT Liu X Bao JJ Thioredoxin-interacting protein induced α-synuclein accumulation via inhibition of autophagic flux: Implications for Parkinson’s disease CNS neuroscience & therapeutics 2017 23 9 717 723 10.1111/cns.12721 28755477 PMC6492710

[26] Li L Ismael S Nasoohi S Sakata K Liao F-F Thioredoxin-interacting protein (TXNIP) associated NLRP3 inflammasome activation in human Alzheimer’s disease brain Journal of Alzheimer’s Disease 2019 68 1 255 265 10.3233/JAD-180814 PMC10947081 30741672

[27] Melone MAB Dato C Paladino S Coppola C Trebini C Verapamil inhibits Ser202/Thr205 phosphorylation of tau by blocking TXNIP/ROS/p38 MAPK pathway Pharmaceutical research 2018 35 2 1 14 10.1007/s11095-017-2276-2 29404777

[28] Jo S Chen J Xu G Grayson TB Thielen LA miR-204 controls glucagon-like peptide 1 receptor expression and agonist function Diabetes 2018 67 2 256 264 10.2337/db17-0506 29101219 PMC5780066

[29] Xu W von Strauss E Qiu C Winblad B Fratiglioni L Uncontrolled diabetes increases the risk of Alzheimer’s disease: a population-based cohort study Diabetologia 2009 52 6 1031 10.1007/s00125-009-1323-x 19280172

[30] Femminella GD Edison P Evaluation of neuroprotective effect of glucagon-like peptide 1 analogs using neuroimaging Alzheimer’s & Dementia 2014 10 S55 S61 10.1016/j.jalz.2013.12.012 24529526

[31] Perry T Greig NH Enhancing central nervous system endogenous GLP-1 receptor pathways for intervention in Alzheimer’s disease Current Alzheimer Research 2005 2 3 377 385 10.2174/1567205054367892 15974903

[32] Liang C Zhu H Xu Y Huang L Ma C MicroRNA-153 negatively regulates the expression of amyloid precursor protein and amyloid precursor-like protein 2 Brain research 2012 1455 103 13 10.1016/j.brainres.2011.10.051 22510281

[33] Tiribuzi R Crispoltoni L Porcellati S Di Lullo M Florenzano F miR128 up-regulation correlates with impaired amyloid β (1–42) degradation in monocytes from patients with sporadic Alzheimer’s disease Neurobiology of aging 2014 35 2 345 356 10.1016/j.neurobiolaging.2013.08.003 24064186

[34] Lee K Kim H An K Kwon O-B Park S Replenishment of microRNA-188–5p restores the synaptic and cognitive deficits in 5XFAD Mouse Model of Alzheimer’s Disease Scientific reports 2016 6 1 1 14 10.1038/srep34433 27708404 PMC5052619

[35] Wang X Tan L Lu Y Peng J Zhu Y MicroRNA-138 promotes tau phosphorylation by targeting retinoic acid receptor alpha FEBS letters 2015 589 6 726 729 10.1016/j.febslet.2015.02.001 25680531

[36] He D Tan J Zhang J miR-137 attenuates Aβ-induced neurotoxicity through inactivation of NF-κB pathway by targeting TNFAIP1 in Neuro2a cells Biochemical and biophysical research communications 2017 490 3 941 947 10.1016/j.bbrc.2017.06.144 28655611

[37] Souza VC Morais GS Jr Henriques AD Machado-Silva W Perez DIV Whole-blood levels of MicroRNA-9 are decreased in patients with late-onset Alzheimer disease American Journal of Alzheimer’s Disease & Other Dementias® 2020 35 1533317520911573 10.1177/1533317520911573 PMC10623914 32301334

[38] Giuliani A Gaetani S Sorgentoni G Agarbati S Laggetta M Circulating Inflamma-miRs as Potential Biomarkers of Cognitive Impairment in Patients Affected by Alzheimer’s Disease Frontiers in Aging Neuroscience 2021 13 647015 10.3389/fnagi.2021.647015 33776746 PMC7990771

[39] McKhann GM Knopman DS Chertkow H Hyman BT Jack CR Jr The diagnosis of dementia due to Alzheimer’s disease: recommendations from the National Institute on Aging-Alzheimer’s Association workgroups on diagnostic guidelines for Alzheimer’s disease Alzheimer’s & dementia 2011 7 3 263 269 10.1016/j.jalz.2011.03.005 PMC3312024 21514250

[40] Morris JC Clinical dementia rating: a reliable and valid diagnostic and staging measure for dementia of the Alzheimer type International psychogeriatrics 1997 9 S1 173 176 10.1017/s1041610297004870 9447441

[41] Scheltens P Launer LJ Barkhof F Weinstein HC van Gool WA Visual assessment of medial temporal lobe atrophy on magnetic resonance imaging: interobserver reliability Journal of neurology 1995 242 9 557 560 10.1007/BF00868807 8551316

[42] Scheltens P Erkinjunti T Leys D Wahlund L-O Inzitari D White matter changes on CT and MRI: an overview of visual rating scales European neurology 1998 39 2 80 89 10.1159/000007921 9520068

[43] Folstein MF Folstein SE McHugh PR “Mini-mental state”: a practical method for grading the cognitive state of patients for the clinician” Journal of psychiatric research 1975 12 3 189 198 10.1016/0022-3956(75)90026-6 1202204

[44] Li M Rai AJ DeCastro GJ Zeringer E Barta T An optimized procedure for exosome isolation and analysis using serum samples: application to cancer biomarker discovery Methods 2015 87 26 30 10.1016/j.ymeth.2015.03.009 25814440

[45] Mustapic M Eitan E Werner JK Jr Berkowitz ST Lazaropoulos MP Plasma extracellular vesicles enriched for neuronal origin: a potential window into brain pathologic processes Frontiers in neuroscience 2017 11 278 10.3389/fnins.2017.00278 28588440 PMC5439289

[46] Livak KJ Schmittgen TD Analysis of relative gene expression data using real-time quantitative PCR and the 2−ΔΔCT method methods 2001 25 4 402 408 10.1006/meth.2001.1262 11846609

[47] Thompson AG Gray E Heman-Ackah SM Mäger I Talbot K Extracellular vesicles in neurodegenerative disease—pathogenesis to biomarkers Nature Reviews Neurology 2016 12 6 346 357 10.1038/nrneurol.2016.68 27174238

[48] Jain G Stuendl A Rao P Berulava T Centeno TP A combined miRNA–piRNA signature to detect Alzheimer’s disease Translational psychiatry 2019 9 1 1 12 10.1038/s41398-019-0579-2 31591382 PMC6779890

[49] Goetzl EJ Boxer A Schwartz JB Abner EL Petersen RC Altered lysosomal proteins in neural-derived plasma exosomes in preclinical Alzheimer disease Neurology 2015 85 1 40 47 10.1212/WNL.0000000000001702 26062630 PMC4501943

[50] Walsh DM Cha DJ Mengel D Mustapic M Liu W miR-212 and miR-132 are down- regulated in neurally-derived plasma exosomes of Alzheimer’s patients Frontiers in neuroscience 2019 13 1208 10.3389/fnins.2019.01208 31849573 PMC6902042

[51] Serpente M Fenoglio C D’Anca M Arcaro M Sorrentino F MiRNA Profiling in Plasma Neural-Derived Small Extracellular Vesicles from Patients with Alzheimer’s Disease Cells 2020 9 6 1443 10.3390/cells9061443 32531989 PMC7349735

[52] Hornung S Dutta S Bitan G CNS-Derived Blood Exosomes as a Promising Source of Biomarkers: Opportunities and Challenges Frontiers in Molecular Neuroscience 2020 13 38 10.3389/fnmol.2020.00038 32265650 PMC7096580

[53] Schneider R McKeever P Kim T Graff C Van Swieten JC Downregulation of exosomal miR-204-5p and miR-632 as a biomarker for FTD: a GENFI study Journal of Neurology, Neurosurgery & Psychiatry 2018 89 8 851 858 10.1136/jnnp-2017-317492 29434051 PMC6045452

[54] Inohara N Ding L Chen S Núñez G harakiri, a novel regulator of cell death, encodes a protein that activates apoptosis and interacts selectively with survival-promoting proteins Bcl-2 and Bcl-XL The EMBO journal 1997 16 7 1686 1694 10.1093/emboj/16.7.1686 9130713 PMC1169772

[55] Zhu X Yu L Tao W Jin J Xu Y O3-01-05: miR-204 attenuates memory deficits in a mouse model of Alzheimer’s disease Alzheimer’s & Dementia 2019 15 P876 P877

[56] Olsson B Lautner R Andreasson U Öhrfelt A Portelius E CSF and blood biomarkers for the diagnosis of Alzheimer’s disease: a systematic review and meta-analysis The Lancet Neurology 2016 15 7 673 684 10.1016/S1474-4422(16)00070-3 27068280

[57] Jack CR Jr Bennett DA Blennow K Carrillo MC Dunn B NIA-AA research framework: toward a biological definition of Alzheimer’s disease Alzheimer’s & Dementia 2018 14 4 535 562 10.1016/j.jalz.2018.02.018 PMC5958625 29653606

[58] Janelidze S Stomrud E Smith R Palmqvist S Mattsson N Cerebrospinal fluid p-tau217 performs better than p-tau181 as a biomarker of Alzheimer’s disease Nature communications 2020 11 1 1 12 10.1038/s41467-020-15436-0 PMC7125218 32246036

[59] Janelidze S Berron D Smith R Strandberg O Proctor NK Associations of Plasma Phospho-Tau217 Levels With Tau Positron Emission Tomography in Early Alzheimer Disease JAMA neurology 2020 78 2 149 156 10.1001/jamaneurol.2020.4201 PMC7653537 33165506

